# Optimal use of lenvatinib in the treatment of advanced thyroid cancer

**DOI:** 10.1186/s41199-017-0026-0

**Published:** 2017-10-24

**Authors:** Shunji Takahashi, Naomi Kiyota, Makoto Tahara

**Affiliations:** 1 Department of Medical Oncology, Cancer Institute Hospital, Japanese Foundation for Cancer Research, 3-8-31 Ariake, Koto-ku, Tokyo, 135-8550 Japan; 2 Department of Medical Oncology and Hematology, Kobe University Hospital, Kobe, Japan; 3 Department of Head and Neck Medical Oncology, National Cancer Center Hospital East, Kashiwa, Japan

**Keywords:** Adverse event, Lenvatinib, Multitargeted kinase inhibitor, Thyroid cancer, Vascular endothelial growth factor receptor

## Abstract

The development of orally active, multitargeted kinase inhibitors (MKIs) represents a significant advance in the treatment of progressive, metastatic thyroid cancer. Lenvatinib, an MKI targeting vascular endothelial growth factor receptor, fibroblast growth factor receptor, platelet-derived growth factor receptor, c-Kit, and RET, has shown efficacy in stabilizing previously progressive disease, with emerging evidence of a possible benefit in terms of overall survival. However, lenvatinib is associated with a side-effect profile similar to those of other MKIs that might affect the outcome of therapy.

The aim of this review is to summarize the clinical efficacy and safety of MKIs in the treatment of advanced thyroid cancer in pivotal phase III trials. Common adverse events that may occur during lenvatinib therapy and their management are discussed, including conditions in which its administration should be temporarily withdrawn and resumed pending resolution of adverse events. We focus on data from a subanalysis of Japanese patients in the SELECT trial and in a post-marketing study in Japan.

We suggest that lenvatinib is a valuable treatment option for advanced differentiated thyroid cancer. Monitoring and careful management of adverse events including supportive care are required to ensure continuation of therapy.

## Background

Thyroid cancer is a common endocrine malignancy that can be divided into three primary histologic types (differentiated, medullary, and anaplastic). These histologic subtypes have different characteristics and prognoses. The initial treatment of differentiated thyroid cancer is surgical resection, thyroid hormone therapy or radioactive iodine in selected cases. Radioactive iodine can be curative in some cases of metastatic disease and thyroid-stimulating hormone-suppressive hormone therapy can prevent disease progression. However, until recently, the treatment options were limited for patients with metastatic disease that did not respond to the above treatments and for those with medullary and anaplastic thyroid cancer.

Several oral multi-targeted kinase inhibitors (MKIs) have now been approved in Japan and other countries for advanced thyroid cancer, based on the results of phase III trials [1–3]. In Japan, sorafenib and lenvatinib are approved for unresectable differentiated as well as medullary and anaplastic thyroid cancer. Vandetanib is approved for unresectable medullary thyroid carcinoma. Cabozantinib is also approved for medullary thyroid cancer in Western countries, but not yet in Japan. These MKIs target the vascular endothelial growth factor receptor (VEGFR) but their distinct kinase inhibition profiles may result in differences in efficacy and adverse events.

Preclinical cancer models have demonstrated that lenvatinib targets VEGFR 1–3, fibroblast growth factor receptors (FGFR) 1–4, platelet-derived growth factor receptor α, c-Kit, and RET [4, 5]. By targeting the VEGF pathway in particular, it exerts antitumor activity by inhibiting angiogenesis. Lenvatinib and other MKIs represent a significant breakthrough in the treatment of advanced thyroid cancer, but the timing of MKI therapy and the selection of individual agents are not well established, given the absence of direct comparisons of the efficacy and safety of MKIs [6].

MKIs have unique safety profiles and significant toxicities may occur. Therefore, a thorough understanding of the adverse events associated with lenvatinib is needed, along with a comprehensive assessment of adverse events and their management, including the need for drug interruption and dose reduction, and potential treatment discontinuation, during lenvatinib therapy. In this review, we describe the efficacy and safety of lenvatinib in the treatment of advanced thyroid cancer based on data from global and Japanese clinical trials, focusing on the management of common adverse events associated with lenvatinib. The optimal use of lenvatinib through the appropriate management of adverse events will also be discussed.

## Clinical efficacy

The efficacy of lenvatinib in major trials is summarized in Table 1. A global, randomized, double-blind, phase III study (SELECT, Study of E7080 Lenvatinib in Differentiated Cancer of the Thyroid) was conducted to compare the effects of lenvatinib (*n* = 261) versus placebo (*n* = 131) in patients with radioiodine-refractory, differentiated thyroid cancer, including those who had no or one prior treatment with a tyrosine kinase inhibitor [2]. The median progression-free survival (18.3 versus 3.6 months; *p* < 0.001) and the overall response rate (64.8% versus 1.5%; *p* < 0.001) were significantly better in the lenvatinib group than in the placebo group.

**Table 1:** Summary of efficacy in clinical trials of lenvatinib in the treatment of thyroid cancer

Study [ref]	Arm	Subtype	N	PFS, months (95% CI)	OS, months (95% CI)	ORR (%)	DCR (%)
Phase II [8]	Lenvatinib	DTC	58	12.6 (9.9–16.1)	-	50	93
Phase II [9]	Lenvatinib	MTC	59	9.0 (7.0-NE)	-	36	80
Phase II (Japan) [10]	Lenvatinib	DTC	25	25.8 (18.4–NE)	31.8 (31.8–NE)	68	100
		MTC	9	9.2 (1.8–NE)	12.1 (3.8–NE)	22	100
		ATC	17	7.4 (1.7–12.9)	10.6 (3.8–19.8)	24	94
Phase III (general population) [2]	Lenvatinib	DTC	261	18.3 (15.1–NE)	NE	64.8	87.7
	Placebo		131	3.6 (2.2–3.7)		1.5	55.7
Phase III (Japanese population) [7]	Lenvatinib	DTC	30	16.5 (7.4-NE)	NE	63.3	90.0
	Placebo		10	3.7 (1.6–9.1)		0.0	60.0

*ATC* anaplastic thyroid cancer; *DCR* disease control rate; *DTC* differentiated thyroid cancer; *MTC* medullary thyroid cancer; *NE*, not estimable; *ORR* overall response rate; *OS* overall survival; *PFS* progression-free survival

In a subgroup analysis of Japanese patients (lenvatinib, *n* = 30; placebo, *n* = 10) in the SELECT trial [7], the median progression-free survival was longer in the lenvatinib group (16.5 months versus 3.7 months; *p* = 0.067) but statistical significance was not reached, presumably because of the relatively small sample size. The overall response rate was greater in the lenvatinib group (63.3% versus 0%; *p* = 0.0004). The subset was not a randomized group and the *p*-value is nominal.

Two phase II studies examined the effects of lenvatinib in patients with either advanced radioiodine-refractory, differentiated or medullary thyroid carcinomas in Western countries, which included those who had received prior chemotherapy or tyrosine kinase inhibitor therapy [8, 9]. The median progression-free survival times in patients with differentiated or medullary thyroid carcinomas were 12.6 months and 9.0 months, while the overall response rates were 50% and 36%, and the disease control rates were 93% and 80%, respectively.

Another phase II study examined the effects of lenvatinib in 51 Japanese patients with either unresectable radioiodine-refractory, differentiated, medullary, or anaplastic thyroid carcinomas [10, 11]. Previous use of sorafenib for differentiated thyroid cancer was allowed. The median progression-free survival times in patients with differentiated, medullary, or anaplastic thyroid carcinomas were 25.8 months, 9.2 months and 7.4 months, and the median overall survival times were 31.8 months, 12.1 months, and 10.6 months, respectively. The overall response rates were 68%, 22%, and 24%, and the disease control rates were 100%, 100%, and 94%, respectively. These data suggest that lenvatinib may induce tumor shrinkage and prevents disease progression in Japanese patients with medullary or anaplastic thyroid cancer in addition to advanced, differentiated thyroid cancer. Currently, lenvatinib has not been approved for medullary or anaplastic thyroid carcinomas outside Japan and clinical studies are underway in Western countries (NCT00784303, NCT02657369).

Sorafenib has also demonstrated benefit in patients with radioiodine-refractory differentiated thyroid cancer with locally advanced or metastatic disease. In a multicenter, double-blind, phase III trial (DECISION) involving 417 patients randomized to sorafenib or placebo who had not previously received tyrosine kinase inhibitor therapy, the primary endpoint of median progression-free survival was 10.8 months versus 5.8 months (HR 0.59, 95% CI 0.45–0.76), respectively [1]. For advanced medullary thyroid cancer, vandetanib was effective in a phase III trial of 331 patients by significantly extending progression-free survival (HR 0.46, 95% CI 0.31–0.69) [3]. Although the median progression-free survival was not reached, it was predicted to be 30.5 months in the vandetanib group versus 19.3 months in the placebo group. A double-blind phase III trial also demonstrated a benefit of cabozantinib in patients with progressive, metastatic medullary thyroid cancer, with a median progression-free survival of 11.2 months versus 4 months for placebo [12].

## Type and management of adverse events during lenvatinib therapy

Various adverse events have been observed during lenvatinib therapy, which are similar to those reported for other MKIs targeting the VEGF signaling pathway [2, 7]. The most common adverse events include hypertension, diarrhea, fatigue, palmar–plantar erythrodysesthesia and proteinuria (Table 2). In this section, we discuss the characteristics of adverse events that may arise during lenvatinib therapy. We also discuss possible strategies to manage these adverse events, including dose reduction, treatment interruption, supportive medications, and treatment discontinuation, which would mirror those used for other MKIs [13]. A starting dose of 24 mg once a day is indicated for lenvatinib treatment and, in the case of an intolerable adverse event, stepwise dose reductions are recommended from 24 mg to 20 mg, 14 mg, and 10 mg, as recommended in phase 2 and 3 studies of lenvatinib [2, 7]. In the protocols of these two trials, a reduction in dose to 8 mg or 4 mg was requested when a patient needed the dose to be reduced to <10 mg. Resumption of lenvatinib at the reduced dose is usually recommended after interruption, but if the dose at the time of interruption was <14 mg, resumption of the same dose should be considered in order to maintain the dose intensity.

**Table 2:** Major adverse events associated with lenvatinib

		Hypertension	Diarrhea	Fatigue	PPE	Proteinuria	Thrombocytopenia
Phase III (general population) (*n* = 261) [2]	Any grade	68	59	59	32	31	9
	Grade ≥ 3	42	8	9	3	10	2
Phase III (Japanese population) (*n* = 30) [7]	Any grade	87	60	60	70	63	47
	Grade ≥ 3	80	0	13	3	20	7

Events are listed in order of the most frequent events of any grade in the general population

Data are shown as incidence (%). PPE, palmar–plantar erythrodysesthesia

The adverse events are described in decreasing order of frequency (any grade) in the SELECT trial [2], with a particular focus on those that occurred in Japanese patients.

### Hypertension

Because VEGF influences vascular resistance through the production of nitric oxide and enhancing angiogenesis, hypertension frequently occurs with VEGF inhibitors, including sorafenib, vandetanib, cabozantinib, and lenvatinib [14, 15]. Hypertension more frequently occurred in Japanese patients in the SELECT trial [7]. Hypertension of any grade occurred in 86.7% of Japanese patients and grade ≥ 3 occurred in 80.0% (Table 2), whereas the occurrence of hypertension in the general population was 67.8% for any grade and 41.8% for grade ≥ 3 [2]. The overall incidence of hypertension in lenvatinib-treated patients was somewhat higher than that reported for sorafenib in the DECISION trial (40.6% for any grade; 9.7% for grade ≥ 3) [1]. The onset of hypertension was relatively early in the SELECT trial, with a median time to onset of 2.3 weeks [16], while it was 8 days in Japanese patients (unpublished data). Interestingly, treatment-emergent hypertension was associated with a 5.9-month median progression-free survival advantage (hazard ratio 0.59 [95% CI 0.39–0.88]; *P* = 0.009). Given the early onset and high rate of development of hypertension, the baseline blood pressure should be <140/90 mmHg and blood pressure should be measured regularly at home and at each clinical visit during the first few weeks of treatment, at least. In the SELECT study, patients with confirmed systolic BP ≥140 mmHg or diastolic BP ≥90 mmHg were administered antihypertensive agents and monitored every 2 weeks. Patients with systolic BP ≥160 mmHg or diastolic BP ≥100 mmHg, despite optimal management, had their treatment dose reduced.

**Fig. 1:**
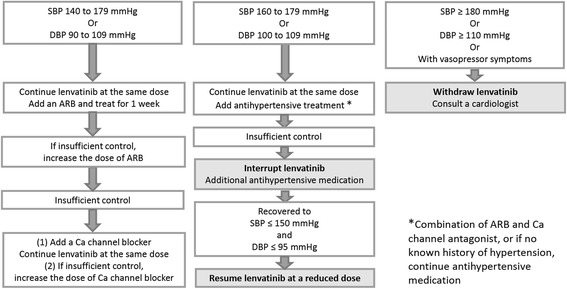
Management of hypertension to enable continuation of lenvatinib treatment. The concomitant use of antihypertensive drugs and a change in the schedule of lenvatinib treatment should be considered according to the severity of hypertension. *Dose escalation is recommended up to a maximum dose for ARB or Ca channel blocker treatment. **A combination of ARB and Ca channel antagonist is recommended

We propose a management plan for hypertension during lenvatinib therapy (Fig. 1), according to the severity of hypertension. Patients should measure blood pressure at the same time in the morning once a day at home. When an elevated blood pressure is detected, the blood pressure should be re-measured after an interval of 5 min or longer. If the blood pressure remains elevated at re-measurement, an antihypertension medication should be started. An antihypertensive drug should be initiated in patients with blood pressure ≥ 140/90 mmHg or a diastolic blood pressure > 90 mmHg in accordance with the guidelines for the management of hypertension [17–19]. Angiotensin II receptor blockers, angiotensin-converting enzyme inhibitors and calcium channel blockers are commonly used to treat MKI-induced hypertension. Although there is little evidence to suggest the superiority of one agent over another in this setting, we recommend the use of an angiotensin II receptor blocker or angiotensin-converting enzyme inhibitor as first-line treatment because of their renoprotective effect. If monotherapy is insufficient in lowering blood pressure, we consider dose escalation of the antihypertensive drug or initiating combination therapy with a calcium channel blocker.

If the systolic blood pressure is ≥160 mmHg or diastolic blood pressure is ≥100 mmHg despite antihypertensive therapy, lenvatinib treatment should be interrupted until a reduction in blood pressure is seen and lenvatinib can be then resumed at a reduced dose.

### Diarrhea

Diarrhea is a manifestation of gastrointestinal toxicity and is common with all MKIs. The incidence of any grade of diarrhea was 60% in Japanese patients in the SELECT trial (Table 2) [7]. Diarrhea was generally mild, of grade 1 or 2 in severity. The median time to first onset was 12.1 weeks [20]. Supportive therapy is needed to prevent dehydration, and an antidiarrheal agent such as loperamide should be considered. Diphenoxylate and atropine (Lomotil) and budesonide are also useful treatments for diarrhea (not approved in Japan). Treatment interruption is required for grade 3 or 4 diarrhea and subsequent lenvatinib dose reductions may be necessary after resuming lenvatinib. In patients administered a diuretic for the management of hypertension, care should be taken to avoid dehydration.

### Fatigue

Fatigue caused by suppression of thyroid function has been reported in clinical studies of other VEGFR inhibitors. Hypothyroidism has been reported in sorafenib-treated patients with renal cancer and in sunitinib-treated patients with gastrointestinal stromal cancer [21, 22]. Additionally, thyroid hormone replacement therapy improved fatigue in axitinib-treated patients with cancer [23]. Adjustment of the thyroid hormone dose is important in patients who experience fatigue, particularly in those with differentiated thyroid cancer for the maintenance of TSH suppression. Destructive thyroiditis and inhibition of VEGFR were proposed as possible mechanisms of thyroid dysfunction associated with tyrosine kinase inhibitors [24].

The incidence of fatigue in Japanese patients in the SELECT trial was relatively high, being 60% for any grade of fatigue and 13.3% for grade ≥ 3 fatigue (Table 2) [7]. Onset of fatigue was seen early after initiation of lenvatinib, with a median time to first onset of 3 weeks [20]. Treatment interruption is recommended if patients experience intolerable fatigue. Severe fatigue can be managed by dose modification or discontinuation. Most patients recovered from fatigue within 1 week after interruption of lenvatinib.

### Palmar–plantar erythrodysesthesia

Palmar–plantar erythrodysesthesia, known as hand–foot syndrome, is characterized by hyperkeratotic, tender lesions on areas of friction/pressure, and usually affects the palms and soles. The cutaneous lesions may be accompanied by paresthesia, tingling and burning sensations of the hands and feet. The incidence of palmar–plantar erythrodysesthesia in Japanese patients in the SELECT trial was higher than that in the general population. The incidence for any grade was 70.0% in Japanese patients and 31.8% in the general population. However, the incidences of grade ≥ 3 were similar (3.3% in Japanese patients and 3.4% in the general population) [2, 7]. The median time to first onset was 5.9 weeks [20].

The severity was grade 2 or less in most cases, enabling continuation of lenvatinib. Compared with the DECISION study of sorafenib, palmar–plantar erythrodysesthesia caused by lenvatinib is less likely to progress to grade ≥ 3. There was no treatment discontinuation due to palmar–plantar erythrodysesthesia in the SELECT study; by contrast, several patients discontinued sorafenib because of palmar–plantar erythrodysesthesia [1].

**Fig. 2:**
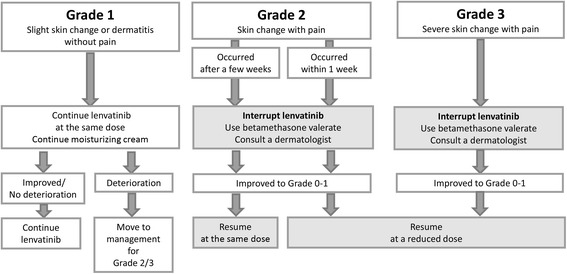
Management of palmar–plantar erythrodysesthesia. Physicians should instruct patients to care for the skin on their hands and feet at the initiation of lenvatinib. The concomitant use of a moisturizing cream and steroid ointments, and a change in the schedule of lenvatinib treatment should be considered according to the severity of palmar–plantar erythrodysesthesia. *Steroid ointments of Group 2 (very strong: e.g., difluprednate) or Group 3 (strong: e.g., betamethasone valerate) are recommended. The treatment regimen and schedule should be determined by a dermatologist

Instructions for skin care of the hands and feet are needed at the initiation of lenvatinib treatment. Preventative measures should be taken, and the condition can be managed by application of topical 20%–40% urea cream and shaving the callouses in most cases (Fig. 2) [25]. A referral to a dermatologist and/or podiatrist may be necessary. If the condition is grade 1 in severity, lenvatinib treatment can be continued at the same dose with use of a moisturizing cream and a hydrocolloid dressing for the feet may be considered [26]. However, severe cases may require dose modification or treatment interruption. If the severity is grade 2, lenvatinib treatment should be interrupted and steroid ointment, such as difluprednate and betamethasone valerate, should be used, followed by consultation with a dermatologist. After recovery to a lower grade, lenvatinib treatment may be resumed at the same dose. If grade 2 occurs within 1 week from initiation of lenvatinib treatment, or if grade 3 occurs in any cases, the incidence suggests that the patient’s skin is sensitive to lenvatinib treatment. Treatment should be interrupted and resumed at the reduced dose after the recovery to grade 0 or 1.

### Proteinuria

In the SELECT trial, the incidence of proteinuria of any grade and grade ≥ 3 was 63.3% and 20.0% in Japanese patients, and 31.0% and 10.0% in the general population, respectively (Table 2) [2, 7]. Proteinuria developed relatively early during lenvatinib therapy, with a median time to first onset of 6.1 weeks. The mechanism underlying proteinuria associated with VEGF inhibitors is unclear. One possible mechanism might be thrombotic microangiopathy, a condition that has been reported for other MKIs [27, 28]. This condition might impair VEGFR-expressing podocytes, which play a central role in glomerular filtration. In addition, because FGF is implicated in podocyte differentiation and recovery after injury, FGF receptor inhibition may adversely affect podocyte function in patients treated with a MKI [29]. However, the relationship between FGF receptor inhibition and toxicity remains unclear, and further investigations are needed.

**Fig. 3:**
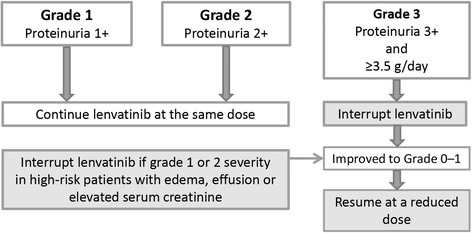
Management of proteinuria to enable continuation of lenvatinib treatment. A change in the schedule of lenvatinib treatment should be considered according to the severity of proteinuria. Careful management of patients with renal dysfunction caused by diabetes or hypertension should be considered. Prescriptions of a diuretic for edema and a statin for hyperlipidemia are recommended

Regular urinalysis should be conducted to detect the onset of proteinuria. Lenvatinib may be continued if proteinuria is grade 1 or 2, based on the criteria in clinical trials [7, 10]. However, if proteinuria is grade 3 or 4 (defined as urinary protein ≥3.5 g/d or a urine protein-to-creatinine ratio ≥ 3.5), or if grade 1 or 2 occurs in high-risk patients with edema, fluid collection or elevated serum creatinine, lenvatinib treatment should be interrupted and spot urine or 24-h urine should be checked to determine urinary protein and/or the urine protein-to-creatinine ratio. Lenvatinib may be continued at the same dose if urinary protein is <3.5 g/day and there is no edema, fluid collection or elevation in serum creatinine. After the proteinuria has recovered or improved to a lower grade, lenvatinib treatment may be re-started at a reduced dose (Fig. 3).

Also, patients with renal dysfunction caused by diabetes or hypertension should be carefully managed. Prescriptions of diuretics for edema and a statin for hyperlipidemia are recommended. In addition, treatment interruption is also recommended if the patient experiences respiratory disorders (i.e., pulmonary edema or effusion), an increase in creatinine, or leg edema of any grade. Its administration may be resumed once these symptoms have improved. Resumption of lenvatinib at a reduced dose is usually recommended after interruption.

Irreversible decline of renal function associated with the VEGFR inhibitor sunitinib has been reported [30, 31], suggesting that VEGFR inhibitors should be discontinued prior to deterioration of proteinuria. Therefore, long-term monitoring for and management of proteinuria is required with lenvatinib treatment.

### Thrombocytopenia

The incidence of thrombocytopenia in Japanese patients in the SELECT trial was 46.7% for any grade and 6.7% for grade ≥ 3, indicating that most cases were of grade ≤ 2, while the incidence in the overall population was 8.8% and 1.5%, respectively (Table 2) [7]. The mechanism of thrombocytopenia in lenvatinib-treated patients is unknown, but it might involve inhibition of c-Kit and FGFR, both of which are involved in megakaryocytopoiesis [32]. Accordingly, inhibition of both pathways is expected to downregulate the production of megakaryocytes and platelets.

Thrombocytopenia increases the risk of bleeding especially in patients with grade 4 thrombocytopenia. Therefore, the platelet count should be carefully monitored. The dose of lenvatinib should be reduced or its administration interrupted in patients with grade 3 or 4 thrombocytopenia. Most cases of thrombocytopenia improved rapidly after drug interruption.

### Hemorrhage

There is a risk of bleeding with all anti-angiogenic MKIs, and it most commonly manifests as epistaxis of mild severity. However, hemorrhage is a common cause of death in patients treated with anti-angiogenic MKIs [33]. Therefore, clinicians should be vigilant for this complication. Clinicians should also pay particular attention to the risk of hemorrhage in patients with thyroid carcinoma, as explained below.

In the SELECT trial, the incidence of any grade epistaxis in Japanese patients was 20%, all of which were grades 1 or 2 [7]. Important information about the risk of bleeding in lenvatinib therapy has emerged from a post-marketing study in Japan (data on file, Eisai Co Ltd.; ClinicalTrials.gov Identifier: NCT02430714), in which 14 cases of hemorrhage were reported (Table 3). Notably, hemorrhage occurred in 3.4% (8 cases out of a total of 238 cases) of patients with anaplastic thyroid carcinoma, which is locally invasive, and in 0.8% (6 cases out of a total of 778 cases) of patients with differentiated thyroid cancer (Table 3). Most cases were associated with tumor shrinkage and necrosis, resulting in bleeding from the tumor or carotid artery (Table 3). The tumor had infiltrated the carotid artery or was located next to it in 12 cases. Therefore, there may be a risk of hemorrhage as a result of arterial invasion or dermal fistula formation, which can lead to serious complications and death.

**Table 3:** Cases of hemorrhage

Patient	Subtype	Type of hemorrhage	Outcome	Time to onset (days)	Dose (mg)	Region infiltrated			Tracheotomy	Fistula formation	Small hemorrhage as a predictor	External radiation
						Carotid artery	Skin	Trachea				
1	DTC	Arterial hemorrhage	Death	37	24	○	○	○	N	Y (skin, trachea, esophagus)	Y (same day)	60 Gy
2	ATC	Arterial hemorrhage	Death	29	20	○	△	△	Y	N	N	N
3	ATC	Arterial hemorrhage	Sequelae	8	24	×	○	○	N	Y (skin)	N	60 Gy
4	ATC	Tumor hemorrhage	Recovered	38	20	○	○	○	N	Y (skin infiltration)	Y (same day), N	60 Gy
		Arterial hemorrhage	Recovered	65	-							
5	ATC	Tumor hemorrhage	Did not recover	20	10	○	○	○	N	Y (skin)	N	40 Gy
6	ATC	Wound hemorrhage	Recovered	19	24	△	×	△	Y	Y (skin)	Y (same day)	N
7	DTC	Venous hemorrhage	Death	106	14	○	○	×	N	Y (skin)	Y (previous day)	N
8	ATC	Arterial hemorrhage	Death	25	24	○	○	○	Y	Y (skin)	Y (5 days before the event previous day)	N
9	DTC	Arterial hemorrhage	Recovered	135	8	△	○	×	N	Y (skin)	N	N
10	ATC	Hemorrhage	Death	153	10	△	×	○	Y	N	Y (a few days before the event	N
11	DTC	Arterial hemorrhage	Death	23	10	○	×	×	Y	N	N	60 Gy
12	DTC	Arterial rupture	Death	209	14	×	×	×	N	Y (trachea, esophagus)	N	60 Gy
13	DTC	Arterial hemorrhage	Death	237	14	△	Unknown	×	N	Y (skin)	N	60 Gy
14	ATC	Arterial hemorrhage	Did not recover	51	24	○	○	○	N	Y (esophagus)	Y (previous day)	N

*ATC* anaplastic thyroid cancer; *DTC* differentiated thyroid cancer; *N* no; *Y*, yes

○The tumor had infiltrated tissues or infiltration was suspected

△The tumor was located next to tissues

×The tumor had not infiltrated tissues and was not located next to tissues

Bleeding occurred within a few months after initiation of lenvatinib in more than half of the cases and may be due to tumor shrinkage, especially in patients with anaplastic thyroid carcinoma. Therefore, diagnostic imaging should be considered monthly for at least the first 3 months of lenvatinib to identify the presence of tumor. In patients with a rapidly growing tumor or metastasis close to the carotid artery, jugular vein or hilus, the administration of MKIs should be carefully evaluated to avoid the risk of hemorrhage [33, 34].

In the above-mentioned post-marketing study, fistula formation occurred in 11 cases. The occurrence of a small hemorrhage as a predictor was observed in seven cases. Seven cases received external radiation. It is uncertain whether radiation therapy should be prioritized over systemic therapy to achieve local disease control. The risk of skin fistula formation must be considered when lenvatinib is combined with radiation therapy. If lenvatinib is to be initiated after radiation therapy to achieve local control, we suggest that lenvatinib should be initiated with caution after radiation therapy to avoid the risk of aerodigestive fistula formation [35, 36]. Hemorrhage was also reported in the area corresponding to a previous radiation field during treatment with the MKI sunitinib in patients with nasopharyngeal carcinoma [33]. The authors of that report proposed that direct vascular invasion by the tumor increased the risk of serious bleeding. Taken together, these findings suggest that vascular-targeting MKIs have a high risk of bleeding and that caution should be taken for disease conditions and treatment history. Instead of MKIs, other systemic therapies such as BRAF-directed therapy for those with a BRAF mutation [37–40] and paclitaxel may be recommended for the patients with a high risk of bleeding.

## Long-term outcomes of lenvatinib treatment and importance of managing adverse events

Until recently, there had been no evidence that MKIs prolonged overall survival. The updated results of the SELECT trial were reported at the 2015 European Cancer Congress and they revealed an improvement in overall survival among patients with radioiodine-refractory differentiated thyroid cancer after adjustment by the rank-preserving structural failure time model [41]. Furthermore, an update of the SELECT trial results reported at the 2016 ASCO Annual Meeting indicated that the median progression-free survival and the median duration of response were 19.4 months and 30 months, respectively [42]. These results suggest that long-term administration of lenvatinib may be beneficial in patients with radioiodine-refractory differentiated thyroid cancer, and that lenvatinib may extend survival in patients with thyroid cancer. However, adverse events are relatively common and must be managed appropriately in order to allow lenvatinib to be administered for a sufficient length of time.

In the SELECT study, liver function tests were performed at baseline, every 2 weeks for 2 months, and monthly thereafter. Renal function, electrolytes, and serum calcium were checked monthly. TSH levels were examined at baseline and monthly. The presence of proteinuria was examined at baseline and periodically during treatment (urine dipstick, if 2+, then 24-h urine collection for measurement of urinary protein and/or urinary protein-to-creatinine ratio). Blood pressure was monitored after 1 week, then every 2 weeks for 2 months, and monthly thereafter. Because blood pressure fluctuates from day to day, frequent management, including self-monitoring by patients if possible, is required, and patients in the SELECT study were also asked to monitor their blood pressure. To monitor disease progression, imaging was performed at 8- to 12-week intervals in the SELECT study.

The approach taken to manage adverse events should consider the type and severity of event, and its potential impact on the patient’s daily life activities [12]. We recommend administration at the same dose in patients with low-grade adverse events, having little impact on treatment continuation, because low-grade adverse events can generally be managed using appropriate supportive strategies and monitoring dependent upon the event types. Such strategies include administration of antihypertensive drugs for hypertension and emollient creams for palmar–plantar erythrodysesthesia. Higher-grade adverse events, however, might require a reduction in the lenvatinib dose and/or temporary treatment interruption until the adverse event has been resolved or its severity declined to an acceptable grade (i.e., to grade 0 or 1). Discontinuation as a possible outcome should also be considered and might be necessary for some patients. However, if the administration of lenvatinib is interrupted temporarily and subsequently resumed once the adverse event has resolved, we recommend administration at a reduced dose upon resuming treatment. Resumption without dose modification may be an option if supportive therapy is available to control the symptoms.

## Summary

Lenvatinib has shown efficacy in stabilizing progressive thyroid cancer, with emerging evidence of a possible benefit in overall survival. However, the use of lenvatinib is associated with adverse events and standard strategies to manage them have not been established. As discussed in this review, adverse events induced by lenvatinib treatment may be managed by strategies that have been employed for other MKIs.

Approaches including dose reduction, treatment interruption, and supportive medications and treatment discontinuation should consider the type and severity of the event, and its potential impact on the patient’s daily activities. By carefully monitoring and managing adverse events, many patients may be able to continue lenvatinib treatment at the appropriate dose.

The combination of lenvatinib with other targeted agents, such as mTOR inhibitors [43–45], is a promising area of investigation, while MEK inhibitors, BRAF inhibitors [37, 38] and immune-oncology agents [46] also show promise for treating radioiodine-refractory, differentiated thyroid cancer. Adverse events may arise with these new agents and combination therapy, and they may warrant a comprehensive and multidisciplinary approach as required in the management of adverse events of lenvatinib.

A thorough understanding of adverse events and their management by patients through communication with their physicians and other medical staff is a key factor in successful long-term treatment with lenvatinib. This in turn leads to improved treatment of cancer patients with other kinase inhibitors showing similar toxicity profiles.

## Conclusion

It is our opinion that the optimal timing of treatment and the management of adverse events are important for the safe and efficacious use of lenvatinib. Accordingly, thorough understanding of adverse events and their management by patients through communication with their physicians is a key factor in successful long-term treatment with lenvatinib.

## Abbreviations

ARB: angiotensin receptor blockers
ASCO: American Society of Clinical Oncology
ATC: anaplastic thyroid cancer
CI: confidence interval
DCR: disease control rate
DTC: differentiated thyroid cancer
FGFR: fibroblast growth factor receptor
HR: hazard ratio
MKI: multitargeted kinase inhibitors
MTC: medullary thyroid cancer
mTOR: mechanistic target of rapamycin
NE: not estimable
ORR: overall response rate
OS: overall survival
PFS: progression-free survival
PPE: palmar–plantar erythrodysesthesia
VEGFR: vascular endothelial growth factor receptor
